# Optical OFDM for SiPM-Based Underwater Optical Wireless Communication Links

**DOI:** 10.3390/s20216057

**Published:** 2020-10-24

**Authors:** Taha Essalih, Mohammad Ali Khalighi, Steve Hranilovic, Hassan Akhouayri

**Affiliations:** 1 Aix Marseille University, CNRS, Centrale Marseille, Institut Fresnel, Marseille, France; Taha.Essalih@fresnel.fr (T.E.); Hassan.Akhouayri@fresnel.fr (H.A.); 2 Department of Electrical & Computer Engineering, McMaster University, Hamilton, ON L8S 4K1, Canada; Hranilovic@mcmaster.ca

**Keywords:** underwater wireless optical communications, optical OFDM, silicon photo-multipliers, dynamic range, clipping noise

## Abstract

Underwater optical wireless systems have dual requirements of high data rates and long ranges in harsh scattering and attenuation conditions. In this paper, we investigate the advantages and limitations of optical orthogonal frequency-division multiplexing (O-OFDM) signaling when a silicon photo-multiplier (SiPM) is used at the receiver in order to ensure high sensitivity. Considering a light-emitting diode (LED) transmitter and taking into account the limited dynamic range imposed by the transmitter and the SiPM receiver, we study the performance of three popular O-OFDM schemes, i.e., DC-biased, asymmetrically-clipped, and layered asymmetrically-clipped O-OFDM (DCO-, ACO-, and LACO-OFDM, respectively). We consider a constraint on transmit electrical power $P_{\mathrm{Txe}}$ and take into account the required DC bias for the three considered schemes in practice, showing the undeniable advantage of ACO- and LACO-OFDM in terms of energy efficiency. For instance, for the considered SiPM and LED components, a spectral efficiency of ∼1 bps/Hz with a data rate of 20 Mbps, a link range of 70 m, and a target bit-error-rate (BER) of $10^{-3}$, ACO and LACO allow a reduction of about 10 and 6 mW, respectively, in the required $P_{\mathrm{Txe}}$, compared to DCO-OFDM. Meanwhile, we show that when relaxing the $P_{\mathrm{Txe}}$ constraint, DCO-OFDM offers the largest operational link range within which a target BER can be achieved. For instance, for a target BER of $10^{-3}$ and a data rate of 20 Mbps, and considering $P_{\mathrm{Txe}}$ of 185, 80, and 50 mW for DCO-, LACO-, and ACO-OFDM, respectively, the corresponding intervals of operational link range are about 81, 74.3, and 73.8 m. Lastly, we show that LACO-OFDM makes a good compromise between energy efficiency and operational range flexibility, although requiring a higher computational complexity and imposing a longer latency at the receiver.

## 1. Introduction

The increasing need to explore underwater resources has given rise to the development of high-performance underwater equipment and robotics with data transmission capability. Underwater wireless data transmission is one of the key features for the efficient operation of such systems. Among the available communication technologies, underwater wireless optical communications (UWOCs) have received increasing attention in the past two decades because of their ability to transmit high data rates with high energy efficiency over short to moderate distances [1,2,3,4,5].

The primary challenges for UWOC systems include extending the range and enhancing the data rate of such links. To address the former, silicon photo-multipliers (SiPMs), also called multi-pixel photon counters (MPPCs), have recently drawn particular attention thanks to their high internal gain, allowing a high receiver (Rx) sensitivity, and hence, operation over large distances. In addition, they offer many implementation and operational advantages over photo-multiplier tubes (PMTs) [6]. On the other hand, the data rate of UWOC links is mainly limited by the limited modulation bandwidth (BW) of the emitting device, which is either a laser diode (LD) or a light-emitting diode (LED), and that of the photo-detector (PD). One efficient approach to deal with channel frequency-selectivity is to use optical orthogonal frequency-division multiplexing (O-OFDM). However, the resulting high signal peak-to-average power ratio (PAPR) can cause a significant performance degradation, given the limited dynamic range (DR) of the transmitter (Tx) opto-electronic components (primarily the emitter and its driver).

Our aim in this paper is to assess the limitation of highly sensitive SiPM receivers when using O-OFDM signaling. For this, we consider the use of LEDs at the Tx, which have the advantage of a relatively high output power and the flexibility of being arranged in arrays. The use of SiPMs at the Rx has the obvious advantage of allowing significant range extension compared to PIN or APD counterparts on the one hand, and operational robustness and implementation simplicity compared to PMTs on the other hand [6]. Given the limited DR of an SiPM, which primarily impacts Rx performance at relatively short ranges [6,7], the use of O-OFDM signaling could impose further limitations in practice. To the best of our knowledge, the performance of an SiPM-based Rx with O-OFDM signaling, taking into account the limited DRs of the Tx and the Rx, has not been investigated in detail so far.

More specifically, we quantify the performance of three O-OFDM techniques from indoor visible-light communication (VLC) systems to underwater settings, namely DC-biased O-OFDM (DCO-OFDM), asymmetrically clipped (ACO-OFDM), and layered ACO (LACO-OFDM). To compare the performances of these schemes, we mostly fix the electrical power at the Tx, as it impacts directly the power consumption of the underwater unit, which is of crucial importance in underwater missions. We take into account the required DC-bias (to minimize the clipping noise) for the three considered schemes, given the LED characteristics. Indeed, the required DC bias for ACO- and LACO-OFDM in practice is commonly neglected in the related theoretical works, which could affect the general conclusions on the choice of the appropriate transmission technique. Taking into account the effects of upper and lower signal clipping at the Tx, SiPM saturation at the Rx at relatively short ranges, and beam attenuation in water, we show that, overall, ACO-OFDM has an undeniable advantage over the two other schemes in terms of energy efficiency for low-to-moderate spectral efficiencies. However, DCO-OFDM allows a higher flexibility (i.e., provides a good tolerance) in terms of operational range when relaxing the constraint on the transmit power. Meanwhile, we show that LACO-OFDM makes a good compromise between energy efficiency and operational range flexibility.

The remainder of this paper is organized as follows. In Section 2, a brief description of the current high spectral efficiency techniques proposed in the UWOC context is presented. Next, Section 3 presents the main assumptions and the mathematical model for the Tx/Rx. A brief presentation of the O-OFDM techniques is presented in Section 4, where the adequacy of the most recent techniques to our application is also discussed. Afterwards, the considered O-OFDM schemes in this work are described briefly in Section 5. Next, a set of numerical results is presented in Section 6 to study the performance of an SiPM-based system. Lastly, Section 7 concludes the paper.

## 2. High Spectral Efficiency State-of-the-Art Techniques

In order to push the data rate beyond the limited BW of the opto-electronic components, two common approaches are serial transmission with channel equalization at the Rx, and the use of high spectral efficiency modulation techniques such as multiple sub-carrier modulation [8]. For instance, the use of on-off keying (OOK), pulse-amplitude modulation (PAM), and pulse-position modulation (PPM) with frequency-domain equalization (FDE) was considered in [9,10]. Several works also have considered the use of O-OFDM for UWOC links, with the advantages of using a simple single-tap equalizer to equalize the frequency-selective aggregate channel. These latter approaches have been primarily applied experimentally in clear underwater environments with a LD Tx and DCO-OFDM signaling. For instance, a data rate of 1.45 Gbps over a range of 4.8 m was reported in [11] based on pre-emphasized O-OFDM symbols and using an avalanche photo-diode (APD) at the Rx, attaining a bit-error-rate (BER) of $9.1\times 10^{-4}$. A similar approach was used in [12] while adjusting the LD bias, where a data rate of 4.8 Gbps with a BER of $2.6\times 10^{-3}$ was reported over 5.4 m. Using a power-loading technique [13] at the Tx and a simple PIN PD with a lens at the Rx, a data rate of 1.3 Gbps over 6 m was achieved in [14] with a BER of $2\times 10^{-3}$. In [15], using power-loading and pre-emphasizing at the Tx and a PIN PD with a lens at the Rx, a 12.4 Gbps link was established over 1.7 m. Furthermore, using an LED and without power-loading, [16] demonstrated a 161 Mbps data rate over 2 m using a PIN PD and a pair of focusing lenses, achieving a BER of $2.5\times 10^{-3}$.

On the other hand, downlink/uplink transmission was considered in [17] over 26 m, including a 5 m air and a 21 m water channel, where a 5.5 Gbps data rate with BER $\approx 2\times 10^{-3}$ was achieved using power-loading at the Tx with an APD at the Rx. This experiment was repeated in [18] using an SiPM with a plano-convex lens at the Rx, where a data rate of 312 Mbps was achieved with a BER of ∼$3\times 10^{-3}$.

As mentioned in the previous section, we consider the three techniques of DCO, ACO, and LACO-OFDM and investigate their suitability for use in an SiPM-based UWOC system.

## 3. General Assumptions

Consider a perfectly aligned link and assume perfect time synchronization between the Tx and the Rx; also, neglect the oceanic turbulence assuming negligible temperature and salinity gradient and sea currents [19,20]. At the Tx side, an LED or an array of LEDs is used as emitting device. Consequently, intensity modulation (IM) is used at the Tx with direct detection (DD) at the Rx. It is also assumed that the Rx has a perfect knowledge of the aggregate channel impulse response (CIR), i.e., including the impulse responses of the Tx, the aquatic channel, and the Rx. The general models of the Tx and the Rx are specified in the following.

### 3.1. Modeling Received Optical Power

Consider the Lambertian model for the LED radiation pattern $P_{t}$, which is given by [21]

(1) $$P_{t}=P_{\mathrm{Tx}}\frac{m+1}{2\pi }\cos^{m}\left(\theta \right),\;\theta \in \left[0,\pi /2\right],$$

where θ is the angle of irradiance of the LED, *m* is the Lambertian order and $P_{\mathrm{Tx}}$ is the transmitted optical power. Ignoring system losses, the received power $P_{\mathrm{Rx}}$ on the PD is then given by [6]:

(2) $$P_{\mathrm{Rx}}=P_{\mathrm{Tx}}\exp\left(-KZ\right)\;\frac{A}{Z^{2}}.$$

Here, *Z* represents the distance between the Tx and the Rx, *K* is the beam diffuse attenuation coefficient (which depends on the wavelength and water turbidity), and *A* denotes the effective active area of the PD. Note that the approximate exponential attenuation, which is considered here for the sake of simplicity, is valid for the case of using a diffuse light source and in low-turbidity waters [22]. Nevertheless, this does not incur any loss of generality for the presented study.

### 3.2. SiPM Modeling

An SiPM is an array of APDs biased at the Geiger mode with the ability to detect a single photon arriving on its surface. These “pixels” are also commonly called single-photon avalanche diodes (SPADs). The photon counting process can be modeled by a Poisson distribution. Denoting the average number of received photons on the SiPM surface by μ, and the number of the counted photons by $C_{\mathrm{ph}}$, the photon count probability $Pr(C_{\mathrm{ph}}=k)$ is given by [7,23]

(3) $$\begin{array}{c} Pr\left(C_{\mathrm{ph}}=k\right)=\exp\left(-\mu \right)\frac{\mu^{k}}{k!}, \end{array}$$

where $C_{\mathrm{ph}}=\sum_{i=1}^{N_{\mathrm{SPAD}}}c_{\mathrm{ph}}\left(i\right)$, with $c_{\mathrm{ph}}\left(i\right)$ the photon count of the $i^{\mathrm{th}}$ SPAD, and $N_{\mathrm{SPAD}}$ the number of SPADs. The average photon count μ can be expressed as a function of the received optical power as [6,7]:

(4) $$\mu =\left(\frac{Y_{\mathrm{PDE}}}{E_{p}}P_{\mathrm{Rx}}+f_{\mathrm{DCR}}\right)\left(1+P_{\mathrm{AP}}+P_{\mathrm{CT}}\right)\;T_{s},$$

where $Y_{\mathrm{PDE}}$ is the photon detection efficiency (which includes the SiPM fill factor), $f_{\mathrm{DCR}}$ denotes the dark count rate, $P_{\mathrm{AP}}$ is the probability of after pulsing, and $P_{\mathrm{CT}}$ stands for the probability of cross talk. $E_{p}$ denotes the photon energy, and $T_{s}$ is the average counting period. Another important parameter of an SiPM is its dead time $\tau_{d}$, which is the time required for each SPAD to recharge after detecting a photon. This causes the “saturation” of the SiPM at relatively high received powers (i.e., short ranges) [23], resulting in a nonlinear distortion (NLD) on the received signals. The dead time depends on the quenching device used in the SiPM design. Here, we consider passive quenching (PQ) devices for which the average output photon count, denoted here by $\mu_{\mathrm{PQ}}$, is given by [7]

(5) $$\mu_{\mathrm{PQ}}=\mu \;\exp\left(-\frac{\mu \;\tau_{d}}{T_{s}\;N_{\mathrm{SPAD}}}\right).$$

## 4. Optical OFDM Signaling

O-OFDM is popular in indoor VLC systems and is proposed as a basis for evolving standards [21,24]. DCO and ACO are among the most popular, while LACO is a recently proposed scheme [25] with a higher computational complexity and imposing more latency on the Rx side. Before focusing on these three, this section provides a brief presentation of the most important O-OFDM signaling schemes proposed in the literature so far.

### 4.1. Classical O-OFDM Schemes

Due to the use of IM/DD signaling, the transmitted signal must be strictly positive and real. Therefore, most proposed O-OFDM schemes impose the Hermitian symmetry constraint in the frequency domain to obtain a real signal in the time domain [26]. In order for DCO-OFDM to ensure unipolarity of the transmitted signal, a DC bias is added to the signal before upper and lower clipping, which results in a relatively high spectral efficiency at the cost of a lower power efficiency. In ACO-OFDM, on the other hand, only the odd sub-carriers are modulated, resulting in a time-domain signal with anti-symmetry property. After applying hard clipping to the negative part, the resulting clipping noise does not affect the modulated sub-carriers. Nevertheless, this incurs a spectral efficiency loss of factor 2, compared to DCO-OFDM, but provides a better power efficiency [8].

An alternative technique to ACO-OFDM is the so-called flip-OFDM [27], also known as U-OFDM [28,29,30], which consists of transmitting the positive amplitude portions of the signal followed by the flipped negative amplitude portions, separated by a cyclic prefix (CP) to avoid interference between the negative and positive blocks. Compared to ACO, the effective Rx noise variance for signal detection is doubled, but the Rx has a lower computational complexity. Another proposed alternative to ACO-OFDM (with the same spectral efficiency), is the so-called pulse-amplitude-modulation discrete-multi-tone (PAM-DMT) [31], by which PAM symbols are converted to imaginary signals before applying the Hermitian symmetry.

### 4.2. Improving Spectral Efficiency with Respect to DCO-OFDM

To improve the spectral efficiency of ACO-OFDM, several techniques have been proposed. In [32], enhanced U-OFDM (eU-OFDM) was proposed, which combines multiple U-OFDM signals at the Tx (by sending them on different “depths”) and performs signal detection at the Rx based on successive interference cancellation to remove the interference between the signals of the different depths. The proposed asymmetrically clipped DCO (ADO) -OFDM [33] uses ACO on odd sub-carriers and DCO signaling on even sub-carriers. The hybrid ACO (HACO) -OFDM [34] is similar to ADO but uses PAM-DMT on even sub-carriers (instead of DCO), where the negative part of the corresponding signals is clipped before transmission. The recently proposed LACO-OFDM consists in a layering of multiple ACO signals, which depends on the signal’s symmetry propeties (see Section 5.3 for more details). It has the advantage of improved spectral efficiency that can approach that of DCO [25]. It also benefits from more flexibility, in the sense that the overall transmission data rate and performance can be adjusted by setting independently the signal constellation size and the allocated power within each layer. Another proposed scheme, called hybrid PAM-DMT (HPAM-DMT) [35], uses a similar concept as LACO, where the transmitted signal is composed of different “groups” of signals: The first group consists of a real PAM-DMT (RPAM-DMT) signal, and the successive groups are obtained from RPAM-DMT modulated signals sent on specific sub-carriers. The advantage of HPAM-DMT over LACO is its lower computational complexity at the Rx, but this comes at the drawback of a lower power efficiency.

A detailed comparison of the above-mentioned techniques can be found in [36,37,38]. In particular, it was shown in [38] that the two-layer LACO and HACO are more power-efficient compared to ADO for a given spectral efficiency. For a spectral efficiency of larger than ∼4 bps/Hz, the four-layer LACO-OFDM outperforms the other proposed hybrid schemes in terms of power efficiency [38].

## 5. Description of the Considered Signaling Schemes

Given the advantages of LACO-OFDM, as explained in the previous section, it is considered in this paper, as well as the popular DCO- and ACO-OFDM. A brief description of these schemes is provided in the following.

### 5.1. DCO-OFDM

**Figure 1:**
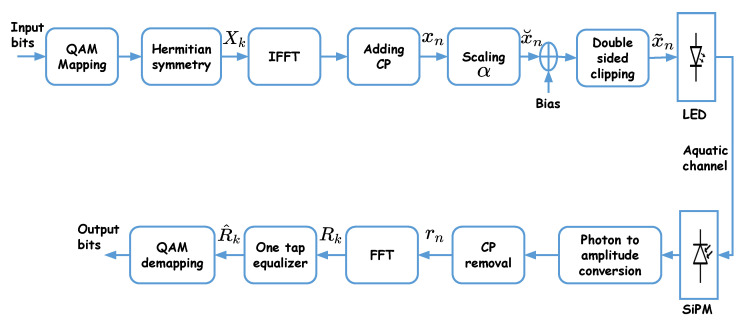
Block diagram of the DCO-OFDM signaling scheme.

Figure 1 shows the block diagram of DCO-OFDM signaling for a typical SiPM-based UWOC link. First, blocks of input data bits are mapped into *M*-QAM (complex) symbols $X_{k}$, k=0,1...,N−1, which are then passed through an inverse fast Fourier transform (IFFT) block generating the “time-domain” OFDM signal $x_{n}$:

(6) $$x_{n}=\frac{1}{\sqrt{N}}\sum_{k=0}^{N-1}X_{k}\exp\left(j\frac{2\pi }{N}nk\right),\;n=0,...,N-1.$$

As explained previously, to ensure that $x_{n}$ is real, Hermitian symmetry is imposed on $X_{k}$ symbols before IFFT such that [26]

(7) $$\left\{\begin{array}{ccc} X_{0} & = & X_{N/2}=0,\\ X_{k} & = & X_{N-k}^{*},\;\;\;\;\;\;0<k<N/2, \end{array}\right.$$

where $.^{*}$ denotes complex conjugate. After IFFT, a CP is added to each block to avoid inter-symbol interference (ISI) as a result of the delay spread $\tau_{0}$ of the aggregate channel. The length of the CP, $N_{\mathrm{CP}}$, is set as to be larger than $\tau_{0}/T_{s}$, where $T_{s}$ is the OFDM symbol duration, hence allowing to restore the signal at the Rx using a one-tap equalizer. Afterwards, a scaling factor α is applied to the signal in order to adequately fit it to the LED DR (this will be further clarified later in Section 5.5); the resulting signal is denoted by ${\overset{˘}{x}}_{n}=\alpha x_{n}$. Next, to obtain a positive signal, a DC bias is added to ${\overset{˘}{x}}_{n}$ before upper and lower clipping due to the limited LED DR, which gives rise to the so called “clipping noise”. The resulting double-side clipped signal is denoted by ${\tilde{x}}_{n}$.

Without loss of generality, we consider driving the LED with a voltage, i.e., $x_{n}$, ${\overset{˘}{x}}_{n}$, and ${\tilde{x}}_{n}$ are all in units of Volts. Note that this is not restricting, as there is a bijection relationship between the input current and voltage of the LED, as described in Section 6.1.

After being transmitted through the aquatic channel, the received optical intensity at the Rx is converted to an electrical signal. “Photon to amplitude conversion” performs the conversion of the number of generated photo-electrons at the SiPM output to an electrical signal amplitude [7]. Here, it is obtained by multiplying $C_{\mathrm{ph}}$ by the photon energy $E_{p}$. Afterwards, after removing the CP, the recovered time-domain OFDM symbols $r_{n}$ are passed through an FFT block whose output is given by

(8) $$R_{k}=\frac{1}{\sqrt{N}}\sum_{n=0}^{N-1}r_{n}\exp\left(-j\frac{2\pi }{N}nk\right),\;k=0,\ldots ,N-1.$$

The obtained signals ${\hat{R}}_{k}$ after equalization of the aggregate channel are then passed to the QAM-demapping block to recover the transmitted bits.

An important point here concerns QAM signal demodulation since the Rx is clearly shot noise limited due to the use of an SiPM. Indeed, for a multi-level modulation scheme, the corresponding signal-dependent noise should be processed carefully, e.g., by performing the so-called square-root transformation [9,39] to avoid a degradation of the Rx performance. However, it has been shown in [39] that for the case of O-OFDM signaling, although the time-domain signal $r_{n}$ at the Rx (before the FFT block in Figure 1) is affected by signal-dependent noise, for the frequency-domain signal (i.e., $R_{k}$ at the output of the FFT block) the noise is in practice nearly independent of the signal [39]. As a result, conventional QAM demodulation can be used in our case, that is, by assuming an effectively signal-independent noise.

Assuming *M*-QAM constellation for $X_{k}$, the spectral efficiency of DCO-OFDM is given by

(9) $$\gamma_{\mathrm{DCO}}=\frac{\log_{2}M\;\left(N-2\right)}{2\;(N+N_{\mathrm{CP}})}\;\left(\mathrm{bps}/\mathrm{Hz}\right).$$

### 5.2. ACO-OFDM

In ACO-OFDM, data are sent only on odd sub-carriers. After applying the Hermitian symmetry, the resulting transmitted frame of symbols (before IFFT) has the following form:

$$\left[\;0,\;X_{0},\;0,\;X_{1},\;0,\ldots ,X_{N/4-1},\;0,\;X_{N/4-1}^{*},\;0,\ldots ,X_{1}^{*}\;\right].$$

This way, after IFFT, the negative part of $x_{n}$ can be clipped without loss of information [40]. The other steps are similar to those described for DCO-OFDM in the previous subsection. Note that in practice, as we will explain later in Section 5.5, a DC bias should still be added to ${\overset{˘}{x}}_{n}$ after adding the CP and scaling, in order to account for the LED I-V characteristics.

Considering an *M*-QAM signal constellation for $X_{k}$, the spectral efficiency of ACO-OFDM is

(10) $$\gamma_{\mathrm{ACO}}=\frac{\log_{2}M\;N}{4\;(N+N_{\mathrm{CP}})}\;\left(\mathrm{bps}/\mathrm{Hz}\right).$$

### 5.3. LACO-OFDM

**Figure 2:**
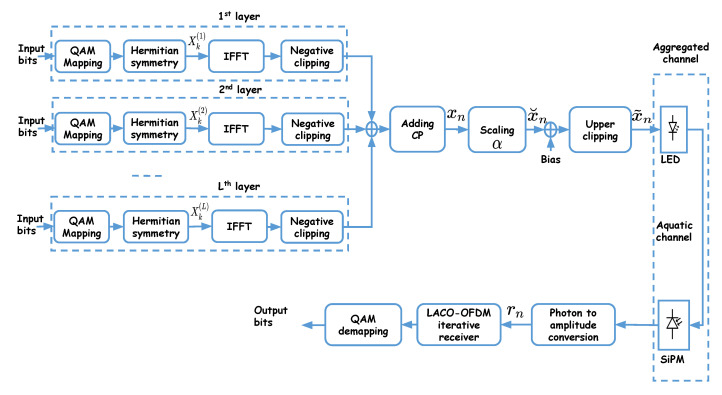
Block diagram of LACO-OFDM signaling with *L* layers.

The general block diagram of LACO-OFDM is shown in Figure 2. The transmitted signal is obtained as the superposition of *L* frames of symbols, each one corresponding to a “layer.” Within each layer, the signaling principle is similar to ACO-OFDM, as explained in the following [25,41].

- For the first layer, ACO-OFDM signaling is used where N/4 symbols and their complex conjugates (according to the Hermitian symmetry requirement) are sent on the odd sub-carriers 2q+1 with q=0,1,..,N/2−1. These are then transformed into time domain after *N* point IFFT.
- For the subsequent layers, the corresponding frames are mapped onto the remaining even sub-carriers.
- For the second layer, N/8 symbols and their complex conjugates are sent on sub-carriers 2(2q+1) with q=0,1,...,N/4−1; they are transformed into time domain after *N*-point IFFT, while the amplitudes of the remaining sub-carriers are set to zero.
- In general, for the $\ell^{\mathrm{th}}$ layer, ℓ>1, $\left(N/2^{\ell +1}\right)$ symbols and their complex conjugates are sent on sub-carriers $2^{\ell -1}\left(2q+1\right)$ with $q=0,1,...,N/2^{\ell +1}-1$. Then, setting the amplitudes of the remaining sub-carriers to zero, they are transformed into time domain after *N*-point IFFT.

Note that the constellation size in each layer can be adjusted to result in a desired overall data-rate $R_{b}$ (in bps). Afterwards, the time-domain signals of the *L* layers are superimposed before adding the CP, applying scaling, adding a bias, and clipping, as shown in Figure 2. Here, lower clipping of the time-domain signal will generate distortion in the frequency domain, which concerns those sub-carriers unused for a given layer [41]. Nevertheless, since this affects the even sub-carriers, only signals of the layers ℓ≥2 are distorted. For a given sub-carrier used in the $\ell^{\mathrm{th}}$ layer, the resulting distortion from all lower layers will be added to the transmitted signal in the frequency domain. For example, the third layer uses sub-carriers 4,12,...,(N/16), which will be affected by distortion from layers 1 and 2.

**Figure 3:**
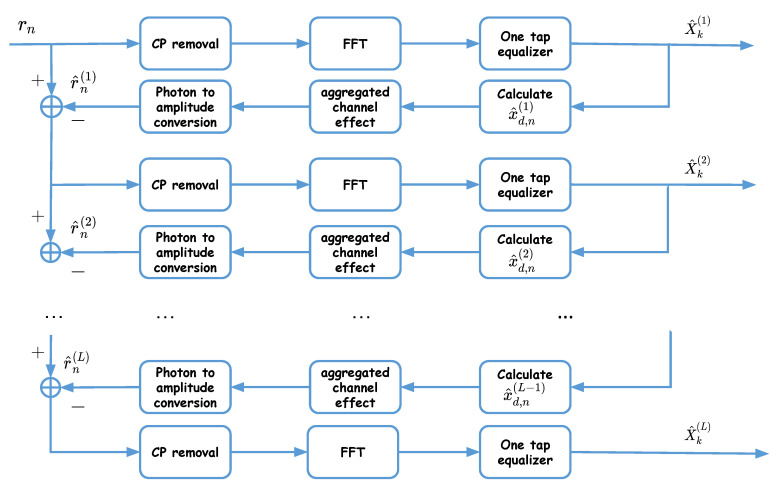
Iterative LACO-OFDM signal detection at the Rx with *L* layers.

To recover the transmitted data, successive detection is done at the Rx, as proposed in [25], where symbols are recovered layer by layer from $r_{n}$, as shown in Figure 3. Note that the first layer is not affected by distortion; hence, the corresponding symbols, ${\hat{X}}_{k}^{\left(1\right)}$, can be detected directly, as in ACO-OFDM, see Section 5.2. These symbols are then used in the second layer to remove the corresponding distortion prior to signal detection. For signal detection in the $\ell^{\mathrm{th}}$ layer, we proceed as follows (see Figure 3):

- Use detected symbols in the previous layers to obtain the corresponding time-domain signals ${\hat{x}}_{d,n}^{\left(1\right)},\cdots ,{\hat{x}}_{d,n}^{\left(\ell -1\right)}$, as it is done at the Tx;
- Calculate their contribution ${\hat{r}}_{n}^{\left(1\right)},\cdots ,{\hat{r}}_{n}^{\left(\ell -1\right)}$ in the received signal;
- Subtract the resulting signals from $r_{n}$;
- Use the same steps as for the first layer on the (partially) distortion-removed signal to obtain ${\hat{X}}_{k}^{\left(\ell \right)}$.

The obtained ${\hat{X}}_{k}^{\left(1\right)},\cdots ,{\hat{X}}_{k}^{\left(L\right)}$ are then passed to a QAM demapper to retrieve the transmitted bits.

As explained, compared to ACO, LACO transmits *L* frames in parallel, which for a given rate allows for using lower-order signal constellations within layers. Hence, it potentially needs a lower signal-to-noise ratio (SNR) for signal detection to achieve a target BER. In other words, LACO would have a better power efficiency, in addition to the advantage of having a lower PAPR [25,42]. Assuming that different signal constellation sizes are used in different layers (to adjust the overall spectral efficiency), i.e., $M_{\ell }$-QAM signal constellation in the $\ell^{\mathrm{th}}$ layer, the spectral efficiency of LACO-OFDM is

(11) $$\gamma_{\mathrm{LACO}}=\frac{N}{2(N+N_{\mathrm{CP}})}\;\sum_{\ell =1}^{L}\frac{1}{2^{\ell }}\log_{2}M_{\ell }\;\left(\mathrm{bps}/\mathrm{Hz}\right).$$

### 5.4. Computational Complexity

From a practical implementation point of view, an important point is the computational complexity of the Tx and the Rx for a given signaling scheme. At the Tx side, DCO and ACO use an *N*-point IFFT with the computational complexity of $O(N\log_{2}\left(N\right))$. For LACO, since an *N*-point IFFT is used for each layer, the computational complexity is on the order of $O(LN\log_{2}\left(N\right))$. However, as for ℓ>1, most of the corresponding symbols are equal to zero, and the computational complexity per layer can be reduced by preforming an $N/2^{\ell -1}$-point IFFT for the $\ell^{\mathrm{th}}$ layer. Then, the overall effective computational complexity can be written as $\left(2-\frac{1}{2^{L-1}}\right)O\left(N\log_{2}\left(N\right)\right))$ [25,42].

At the Rx side, DCO and ACO both need an *N*-point FFT with the computational complexity of $O(N\log_{2}\left(N\right))$. For LACO, an *N*-point FFT is needed per each layer as well as a convolution with the aggregate CIR for ℓ>1. Similar to that explained above, we can perform $N/2^{\ell -1}$-point FFT for ℓ>1. The computational complexity of the convolution is $O(N_{\mathrm{ch}}N)$, where $N_{\mathrm{ch}}$ stands for the approximate length of the aggregate CIR. Overall, the computational complexity of the Rx will be $\left(5-\frac{1}{2^{L-3}}\right)O\left(N\log_{2}\left(N\right)\right))+\left(L-1\right)O\left(N_{\mathrm{ch}}N\right)$ [25].

### 5.5. Adapting the Signal Amplitude to the LED DR

**Figure 4:**
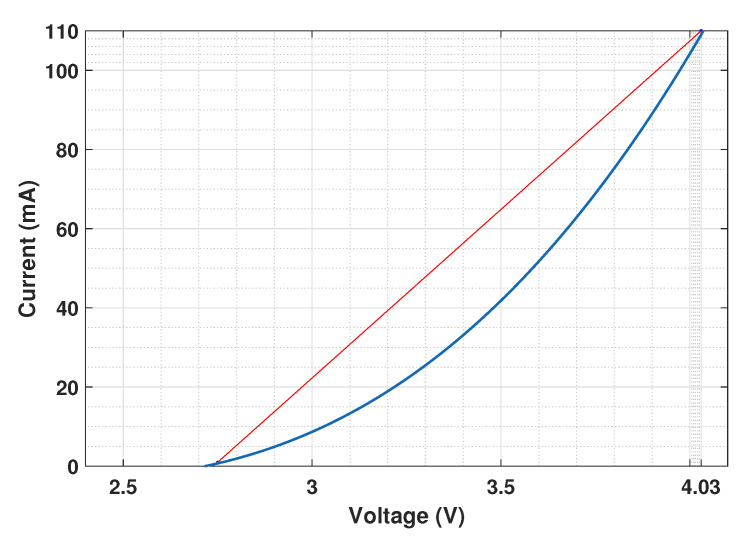
I-V characteristics of the LED [43]. Blue curve: real characteristic; red line: approximate linearized characteristic with $V_{\min}=2.75$ V, $V_{\max}=4.03$ V, $I_{\min}=1$ mA, $I_{\max}=110$ mA.

In practice, before intensity modulation of the LED, the signal needs to be fit to the LED I-V characteristics, or in other words to its DR. Figure 4 shows the I-V characteristics of the LED that we consider in this work [43]. For the sake of simplicity, in our analysis, we ignore the I-V non-linearity within the LED DR and consider the approximate linearized characteristics (the red plot in Figure 4) in the sequel. Note that a digital pre-distortion device can be used to mitigate the non-linear distortion. The study of the effect of non-linear LED characteristics [44,45] is out of the scope of this work.

Firstly, power-normalized QAM constellations are considered, see Figure 1 and Figure 2. It can be shown that the signal $x_{n}$ after IFFT is also power normalized, i.e., $E\left\{x_{n}^{2}\right\}=1$, where E{.} denotes the expected value. After adding the CP, the scale of the signal is changed by multiplying by factor α to obtain ${\overset{˘}{x}}_{n}$. Afterwards, the DC bias, denoted by $B_{\mathrm{DC}}$, is added to it. Denote the lower and upper limits for the LED bias voltage by $V_{\min}$ and $V_{\max}$, respectively. It is obvious that a DC bias of $V_{\min}$ is needed for the cases of ACO- and LACO-OFDM.

Different criteria can be considered for fixing the scaling factor α [46,47]. In particular, for LACO, a different α can be used for each layer. Unless otherwise specified, here it is fixed so as to obtain the same transmit electrical power, $P_{\mathrm{Tx},e}$, for the different considered OFDM schemes in order to make a fair comparison between them. This choice of fixing $P_{\mathrm{Tx},e}$ is justified by the fact that it determines the power consumption of the Tx, which is an essential factor for mobile units (we will further relax this condition and discuss the obtained results in Section 6). Note that for LACO, we use the same α in both layers, although in general it can be adjusted within each layer to further optimize the performance.

$P_{\mathrm{Tx},e}$ primarily depends on the scaling factor α and the DC bias $B_{\mathrm{DC}}$. Having fixed these two parameters, we calculate $P_{\mathrm{Tx},e}$ by averaging the power corresponding to each symbol, which is obtained from its product by the bijective current according to the linearized LED I-V characteristics.

As explained, for the cases of ACO- and LACO-OFDM, $B_{\mathrm{DC}}$ is simply set to $V_{\min}$. For DCO-OFDM, based on a given α, we consider two ways to set the bias, as explained in the following.

Firstly, as a simple method, we use the classical approach of considering the so-called *clipping factor* K [40], which is defined in the following equation, where the effect of the scaling factor is taken into account:

(12) $$B_{\mathrm{DC}}=K\;\sqrt{E\{{\overset{˘}{x}}_{n}^{2}\}}+V_{\min}\approx K\;\alpha +V_{\min}.$$

In the sequel, the clipping factor in decibel is used, i.e., $K_{\mathrm{dB}}=10\;\log_{10}K$.

As a second approach, we consider the idea of [48] to calculate the the optimum value of $B_{\mathrm{DC}}$ so as to minimize the mean square error E between ${\overset{˘}{x}}_{n}$ and double-side clipped signal ${\tilde{x}}_{n}$, while taking $V_{\min}$ and $V_{\max}$ of the LED into consideration:

(13) $$E=E\left\{\sum_{n=0}^{N-1}{\left({\tilde{x}}_{n}-{\overset{˘}{x}}_{n}\right)}^{2}\right\}.$$

For given α, $V_{\min}$, and $V_{\max}$, the optimum bias is calculated numerically by setting $\partial E/\partial B_{\mathrm{DC}}$ to zero. More details are provided in Appendix A.

Unless otherwise specified, we consider the first approach in the presented study, that is, the bias is set by considering a clipping factor $K_{\mathrm{dB}}$ (see Section 6.8).

## 6. Performance Study of the UWOC Link

Considering real characteristics of practical components, a set of numerical results is provided in this section to study the performance of the different O-OFDM techniques.

### 6.1. Parameter Specification

In this work, at the Tx, we consider a NICHIA NSPB510AS LED with emitting wavelength λ=470 nm and 3 dB cut-off frequency of 10 MHz [6]. A Lambertian emission pattern is considered for the LED with m≈45, corresponding to a half-angle of $\approx 10^{\circ }$ [6]. Recall the I-V characteristics of the LED from Figure 4. Without loss of generality, the imperfect conversion efficiency of the LED is neglected. Indeed, an important part of the electrical power consumption at the Tx is due to the conversion efficiency of the LED, typically about 80%, where the corresponding power loss is converted to thermal dissipation. At the Rx, consider a Hamamatsu C13366 3050GA SiPM [49]. The thermal noise effect is reasonably neglected, compared to the SiPM shot noise [50]. The background noise effect is also neglected, assuming that the Rx operates in relatively deep waters [10,51]. Hence, only the shot and dark noises of the SiPM are taken into account. Note that the low DCR of this component is obtained by maintaining the chip temperature at −10 °C by means of a thermoelectric cooler. Thanks to this low DCR, as we will show, the attainable operation ranges are quite larger than those with an older generation of SiPMs, e.g., [52], see for instance the numerical results presented in [6,9].

Lastly, concerning the aquatic channel, the case of clear waters is considered with the diffuse attenuation coefficient K=0.08 m^−1^ [51].

Table 1 contains the main parameters of the Tx, channel, and the Rx used in the simulations.

In the presented study, unless otherwise mentioned, to compare the performances of the different transmission schemes, the same overall link data rate, $R_{b}$, is considered. For DCO and ACO, the considered default signal constellations are 4-QAM and 16-QAM, respectively. For LACO, we will use two layers with, by default, 8-QAM and 4-QAM in the first and the second layers, respectively, which results in the same spectral efficiency of η≈1 bps/Hz as for the two former schemes from (10). Note that no bit or power loading is considered at the Tx in order to better see the limitations of each transmission scheme. Additionally, the number of sub-carriers N=1024 is set by default, which results in an effectively flat channel per sub-channel for the considered data rates. Depending on $R_{b}$, the CP length $N_{\mathrm{CP}}$ is set appropriately (i.e., large enough, compared to the aggregate channel delay spread) in order to avoid ISI. The BW of the aggregate channel is determined by those of the SiPM (∼4 MHz), the LED (∼10 MHz), and the aquatic channel. Lastly, for DCO-OFDM, unless otherwise specified, the DC bias is set by considering a clipping factor of $K_{\mathrm{dB}}=7$ dB, see Section 5.5. This value makes a good compromise between the scaling factor and the clipping noise level for a given $P_{\mathrm{Tx},e}$ (more discussion will be provided later in Section 6.8 and in the Appendix B). For the two other schemes, $B_{\mathrm{DC}}$ is set to $V_{\min}$ of the LED, i.e., 2.75 V, see Figure 4.

### 6.2. Comparison with OOK

**Figure 5:**
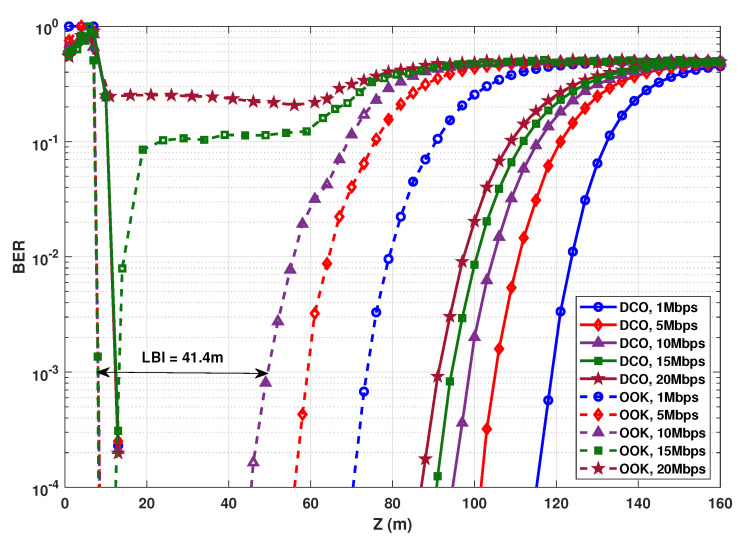
BER comparison of OOK and 4-QAM DCO-OFDM for $P_{\mathrm{Tx},e}=125$ mW. N=1024. $N_{\mathrm{CP}}=2$, 3, 5, 7, and 9, for $R_{b}=1$, 5, 10, 15, and 20 Mbps, respectively.

First of all, in order to elucidate the real interest of O-OFDM signaling, let us compare the performances of DCO-OFDM and OOK modulation schemes for a typical scenario. In Figure 5 BER plots as a function of link distance are presented, considering an electrical transmit power of $P_{\mathrm{Tx},e}=125$ mW. For OOK, the transmitted electrical power corresponding to “Off” symbols is set to 2.75 mW (given $V_{\min}=2.75$ V and $I_{\min}=1$ mA). The transmit power for “On” symbols is set so as to result in an average transmit electrical power of 125 mW. At the Rx side, signal demodulation is based on optimal thresholding, as considered in [9].

First, notice a high BER for very short link ranges (e.g., Z≲8 m for OOK), which is due to SiPM saturation [6,9]. For relatively large link ranges (e.g., Z≳45 m for OOK at $R_{b}=10$ Mbps), the BER reasonably increases due to decreased SNR as a result of channel attenuation.

Similar to [7], we define the low BER interval (LBI, in units of meter) for a given transmission scheme, indicating the interval of link range satisfying a target BER. The LBI can be considered as a measure of link operation flexibility, i.e., the range interval in which the system can function with a low BER. In other words, the larger the LBI, the more flexible is the designed system with respect to the actual link distance. For instance, considering OOK modulation with $R_{b}=10$ Mbps and a target BER of $10^{-3}$, the LBI is around 41.4 m, as indicated in the figure.

As expected, for increased $R_{b}$, the BER performance degrades due to the decreased SNR as a result of shorter symbol duration, and also (for the case of OOK) due to increased ISI. For instance, for BER $=10^{-3}$, the maximum transmission distance $Z_{\max}$ is around 73.7, 59.2, and 49.5 m for OOK with $R_{b}=1$, 5, and 10 Mbps, respectively. For $R_{b}=20$ Mbps that is much higher than the aggregate channel BW, the link with OOK effectively becomes nonoperational. Obviously, DCO-OFDM is robust against channel frequency selectivity, and even for $R_{b}=20$ Mbps we obtain a relatively large $Z_{\max}$. On the other hand, the BER performance is affected more by SiPM saturation due to the higher PAPR and the more complex waveform compared to OOK, which explains the saturation limit at slightly larger distances (i.e., around 12 m at BER $=10^{-3}$). Nevertheless, DCO remains quite advantageous in terms of LBI. For instance, for $R_{b}=1$ Mbps, the LBI is ∼107 m, in contrast to ∼65.5 m for OOK. LBI and $Z_{\max}$ for the different $R_{b}$ values are summarized in Table 2.

### 6.3. Clipping Effect on the Link Performance

**Figure 6:**
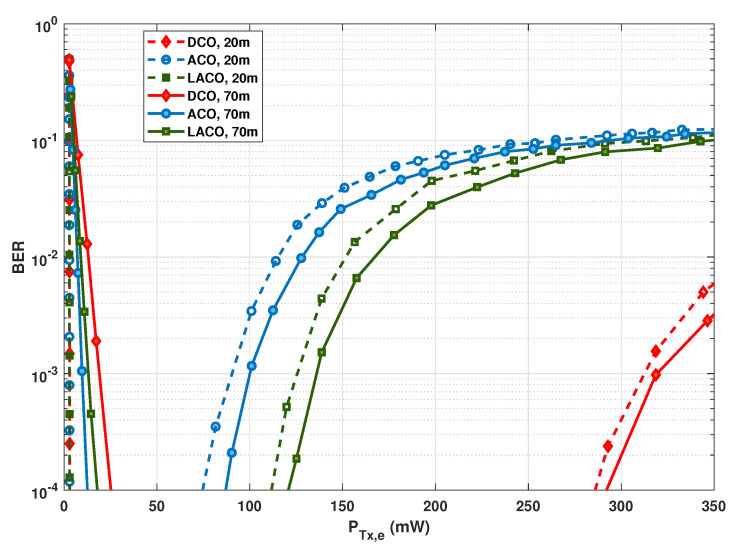
Contrasting clipping effect on DCO-, ACO-, and LACO-OFDM BER performances for Z=20 and 70 m. $R_{b}=20$ Mbps, N=1024, $N_{\mathrm{CP}}=9$.

Let us now study the impact of signal clipping on the performance of DCO-, ACO-, and LACO-OFDM schemes. For this, the spectral efficiency for the three schemes is fixed by setting accordingly the QAM modulation orders, as explained previously in Section 6.1. Figure 6 contrasts the BER performances of these schemes as a function of $P_{\mathrm{Tx},e}$. The link data rate is fixed to 20 Mbps with $T_{s}=5.165\times 10^{-5}$ s, and the link distance is fixed to Z=20 and 70 m, corresponding to relatively moderate and high channel attenuations, respectively.

From Figure 6, for relatively low transmit powers, i.e., $P_{\mathrm{Tx},e}≲25$ mW, notice the close performances of the three techniques, in particular for the relatively short range of Z=20 m. For Z=70 m, ACO and LACO outperform DCO, with a slight advantage for ACO. For instance, at a target BER of $10^{-3}$, the required $P_{\mathrm{Tx},e}$ is around 9.7, 13.2, and 19.1 mW for ACO, LACO, and DCO schemes, respectively.

Meanwhile, the performances of these schemes are affected differently for relatively large transmit powers. For instance, for Z=20 m, upper signal clipping limits the link performance for $P_{\mathrm{Tx},e}$ larger that about 90.4, 125.6, and 310 mW for ACO, LACO, and DCO cases, respectively. The advantage of DCO can be explained by the smaller modulation size used, which results in a lower signal PAPR, and hence a less detrimental effect of the clipping noise. For LACO, which has a lower PAPR than ACO, the clipping effect appears for a smaller $P_{\mathrm{Tx},e}$; yet, we again notice a neat advantage of DCO.

**Figure 7:**
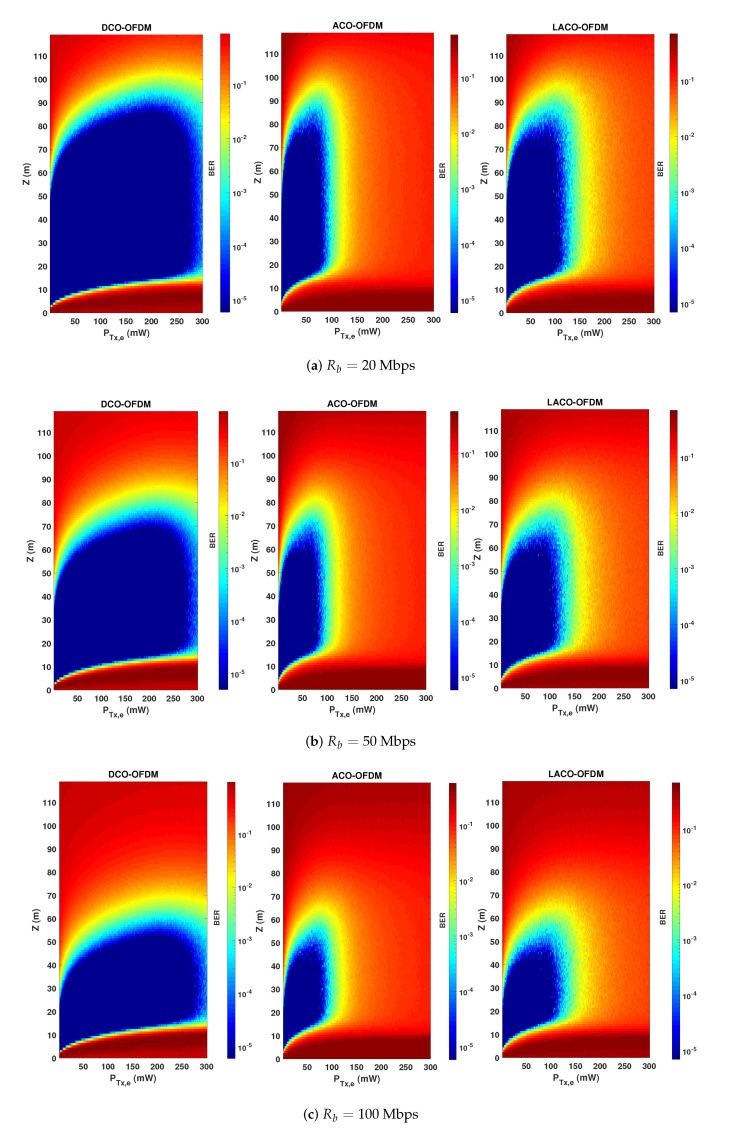
Comparison of BER performance versus the link range *Z* and the transmit electrical power $P_{\mathrm{Tx},e}$ of the three transmission schemes taking into account the LED DR and signal clipping. N=1024, $N_{\mathrm{CP}}=9$, 20, and 28, for data rates of (**a**) 20, (**b**) 50, and (**c**) 100 Mbps, η≈1 bps/Hz.

Notice that, as explained previously, here a non-zero bias is considered for ACO and LACO due to the LED characteristics. The presented results show how these schemes are compared in practice. In particular, for relatively low $P_{\mathrm{Tx},e}$, there is evidence of higher energy efficiency of ACO and LACO over DCO: this latter suffers from the higher DC-bias used. Meanwhile, benefiting from a lower PAPR, DCO is more robust than the two other schemes with respect to increasing the transmit power. Note that, in addition to having a lower PAPR, DCO uses a smaller α. For instance, for $P_{\mathrm{Tx},e}=25$ mW, α is set to 0.0455, 0.153, and 0.092, for DCO, ACO, and LACO-OFDM schemes, respectively. It is worth focusing on the difference between the upper limits of $P_{\mathrm{Tx},e}$ for Z=20 and 70 m in Figure 6. One would expect the same limit irrespective of *Z*, since clipping affects the signal at the Tx side. However, the SiPM saturation at the Rx also affects the link performance for relatively high $P_{\mathrm{Tx},e}$. Consequently, we notice a lower limit for $P_{\mathrm{Tx},e}$ for a smaller *Z*, where the Rx saturation becomes increasingly important. In order to better contrast the performances of the three signaling schemes, while taking into account the effects of signal clipping, channel attenuation, and Rx saturation, we have presented color maps of BER versus $P_{\mathrm{Tx},e}$ and *Z* in Figure 7 for $R_{b}=20$, 50, and 100 Mbps. (For the sake of completeness and following the computational complexity analysis presented in Section 5.4, for the considered LED and SiPM models, $N_{\mathrm{ch}}$ approximately equals 5, 10, and 20 for data rates of 20, 50, and 100 Mbps and the spectral efficiency of η≈1 bps/Hz.)

Considering a target BER of $10^{-3}$, we notice a larger link span for ACO and LACO, compared with DCO for relatively small transmit powers, whereas DCO-OFDM offers the largest LBI. On the other hand, for relatively large $P_{\mathrm{Tx},e}$, as already noticed in Figure 6, DCO undeniably shows more robustness due to having a lower PAPR and a lower sensitivity to Rx saturation, and in this sense can be considered as a more flexible transmission scheme. LACO provides a larger LBI, as compared with ACO, and more flexibility of setting the transmit power. In fact, ACO and LACO use a low DC bias and are penalized by their higher PAPR for high transmit powers. DCO, on the other hand, uses a larger DC bias and a smaller scaling factor for a given $P_{\mathrm{Tx},e}$. As a result, the clipping noise limits its performance for much higher transmit powers.

### 6.4. Impact of Data Rate and Transmit Power

**Figure 8:**
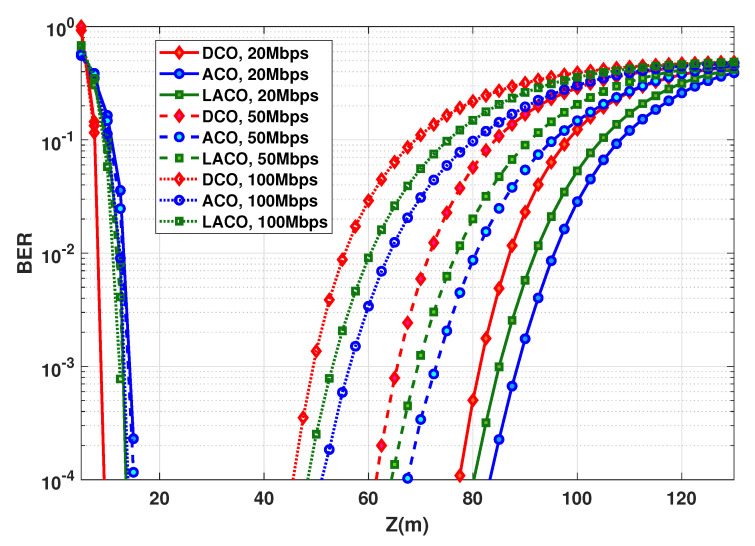
BER performance as a function of distance for DCO-, ACO-, and LACO-OFDM. $P_{\mathrm{Tx},e}=50$ mW, N=1024; $N_{\mathrm{CP}}=9$, 20, and 28, for $R_{b}=20$, 50, and 100 Mbps, respectively.

Consider the BER performance as a function of link distance *Z* for different $R_{b}$. This latter is fixed by setting the OFDM symbol duration $T_{s}$, while keeping *N* unchanged. From Figure 7, to have a sufficiently large LBI for all schemes and the considered bit rates, $P_{\mathrm{Tx},e}$ is set to 50 mW. BER plots are presented in Figure 8, where we notice the best performance for ACO with the largest attainable link span, e.g., around 89 m for $R_{b}=20$ Mbps and a BER of $10^{-3}$ (the corresponding LBI and $Z_{\max}$ are summarized in Table 3). Thus, ACO is the most power-efficient scheme. This confirms the results previously presented in Figure 6 showing that ACO needs a lower $P_{\mathrm{Tx},e}$ to get a target BER, compared to DCO and LACO. We further notice that, for all schemes, LBI shrinks when increasing the data rate, which is due to the decrease in $T_{s}$, i.e., a shorter duration for collecting photons, see Equation (4).

### 6.5. Relaxing the Transmit Power Constraint

**Figure 9:**
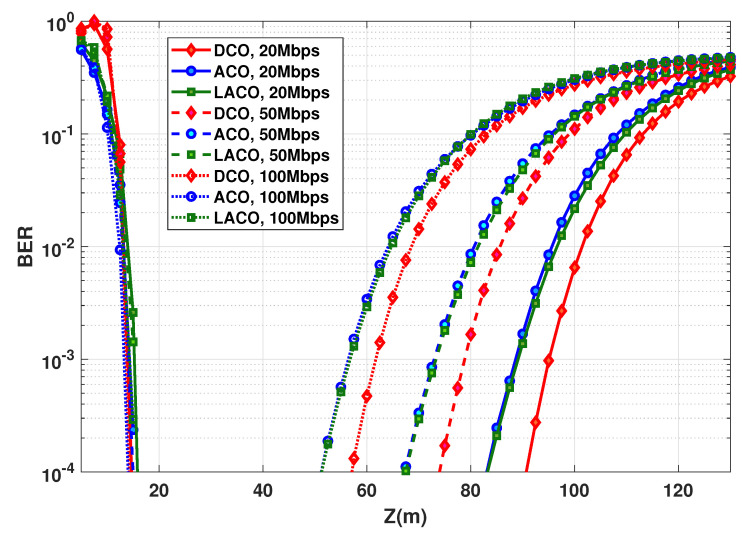
BER as a function of *Z* for ACO-, LACO-, and DCO-OFDM, with $P_{\mathrm{Tx},e}=50$, 80, and 185 mW, respectively. $R_{b}=20$, 50, and 100 Mbps; *N* and $N_{\mathrm{CP}}$ as in Figure 8, η≈1 bps/Hz.

So far, our study was mainly based on the assumption of constrained transmit power, considering the Tx energy efficiency as the main criterion. Consider now as objective the maximization of the LBI in order to obtain the most flexible link operation, while relaxing the requirement of energy efficiency. Accordingly, considering a data rate of 20 Mbps and based on the results of Figure 7, we set $P_{\mathrm{Tx},e}$ to 50, 80, and 185 mW for ACO, LACO, and DCO, respectively, which allow us to maximize the LBI for each scheme. The BER performances are contrasted in Figure 9, where it is seen that DCO provides the best performance, as it could be expected.

The corresponding LBIs and $Z_{\max}$ are summarized in Table 4 for a target BER of $10^{-3}$.

In the sequel, we again fix $P_{\mathrm{Tx},e}$, considering as the main criterion the link energy efficiency.

### 6.6. Impact of QAM Constellation Size

Up to now, the spectral efficiency of ∼1 bps/Hz was considered, corresponding to the signal constellations of 4-QAM for DCO, 16-QAM for ACO, and 8-QAM and 4-QAM for layers 1 and 2 for LACO. Let us now consider larger signal constellations to see how these schemes are affected by a consequently increased PAPR. Accordingly, the constellation is set to 16-QAM for DCO and 256-QAM for ACO, resulting in a spectral efficiency of ∼2 bps/Hz. For LACO, 64-QAM is considered for the first layer and 16-QAM for the second layer. Note that using larger constellation sizes allows us to increase the data rate, while keeping the same OFDM symbol duration $T_{s}$. Nevertheless, in order to make a fair comparison with the previous case (i.e., η≈1 bps/Hz), the same $R_{b}$ values as before are considered, i.e., 20, 50, and 100 Mbps, and accordingly $T_{s}$ is multiplied by 2.

**Figure 10:**
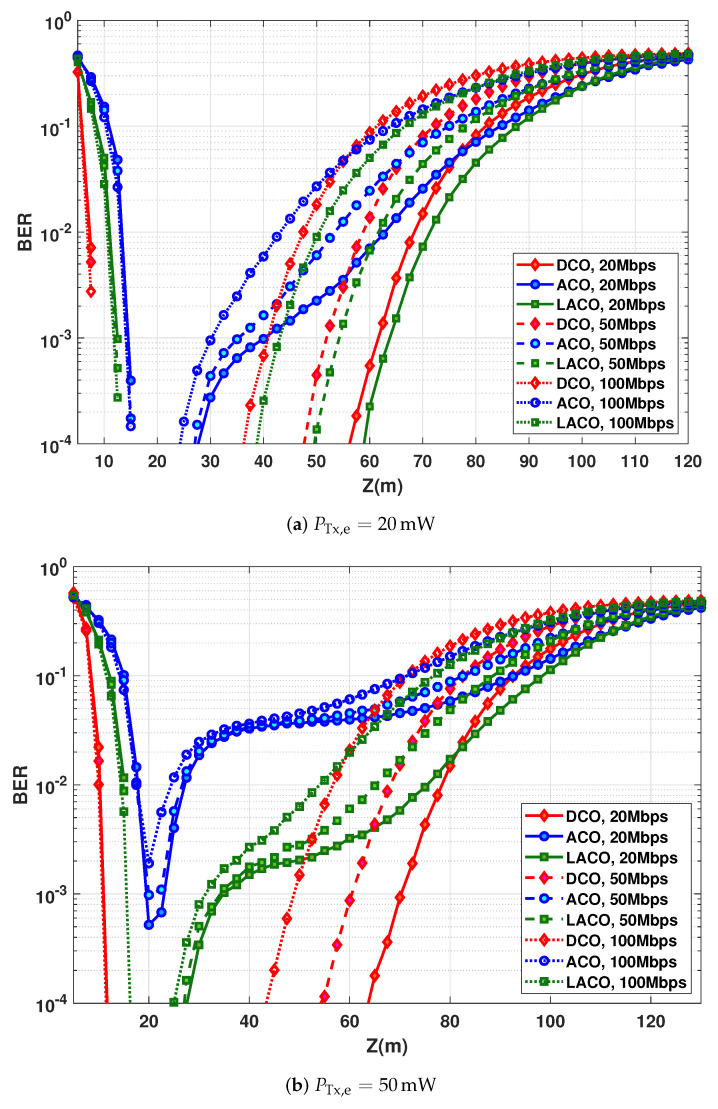
BER performance as a function of distance for DCO, ACO and LACO-OFDM. Spectral efficiency of ∼2 bps/Hz using, e.g., 16QAM for DCO. *N* = 1024, *N*_CP_ = 5, 11, and 20, for *R_b_* = 20, 50, and 100 Mbps, respectively. Electrical transmit power of (**a**) 20 mW and (**b**) 50 mW.

The BER performances versus *Z* are compared in Figure 10 for $P_{\mathrm{Tx},e}=20$ and 50 mW. First, notice from Figure 10a that (as it was the case in Figure 8) for relatively short *Z* where the performance is limited by SiPM saturation, we have a shorter saturation range for DCO. This is due to the high PAPR of ACO and LACO because of using a larger signal constellation. As expected, the best LBI is obtained with DCO for a given target BER. For relatively large *Z*, the lower power efficiency of DCO becomes penalizing. Interestingly, LACO offers the best performance.

**Figure 11:**
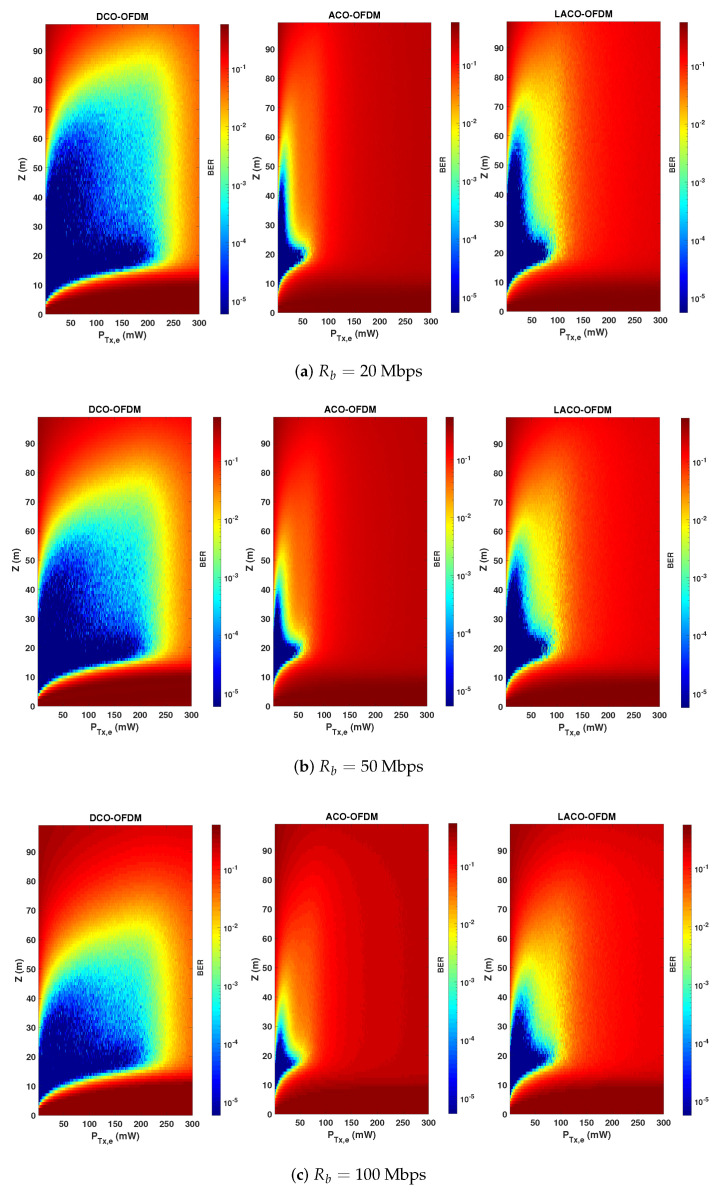
Comparison of BER versus the link range *Z* and the transmit electrical power *P*_Tx,e_ of the three transmission schemes. *N* = 1024, *N*_CP_ = 5, 11, and 20, for data rates of (**a**) 20, (**b**) 50, and (**c**) 100 Mbps, *η* ≈ 2 bps/Hz.

For $P_{\mathrm{Tx},e}=50$ mW, from Figure 10b, notice the superiority of DCO compared to the two other schemes. Indeed, given the large constellation sizes used for ACO and LACO, this transmit power of 50 mW appears to be too high, resulting in considerable signal clipping. Indeed, it was already the case in Figure 10a for ACO. To better understand this limitation, we have presented in Figure 11 color maps of BER versus *Z* and $P_{\mathrm{Tx},e}$ for $R_{b}=20$, 50, and 100 Mbps, where we can notice the significant reduction of the LBI, in particular, for ACO and LACO, as compared with Figure 7. Table 5 summarizes the LBI and $Z_{\max}$ for different data-rates and the target BER of $10^{-3}$ for the three schemes.

It is worth mentioning that here we have considered only two layers for LACO. In fact, using more layers, we can reduce the constellation size in the first layer but at the cost of increased computational complexity. By increasing the number of layers, the spectral efficiency of LACO approaches that of DCO.

### 6.7. Increasing Link Span Using Multiple LEDs

**Figure 12:**
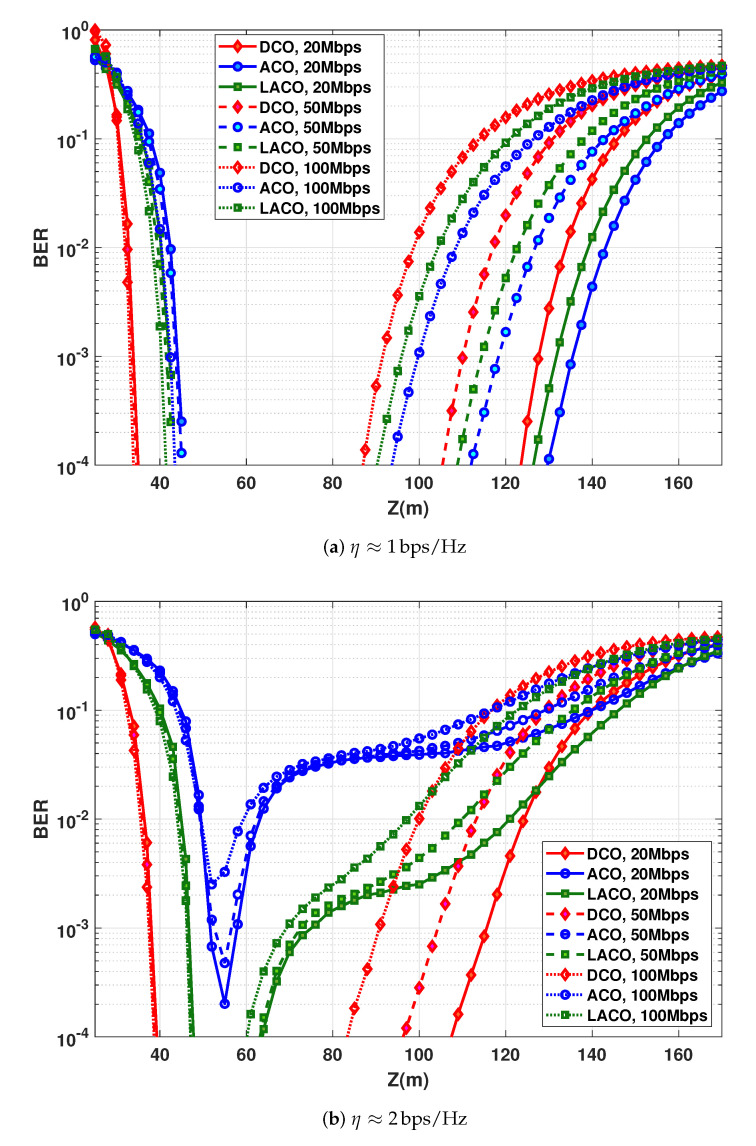
BER performance as a function of distance using 100 LEDs with $P_{\mathrm{Tx},e}=50$ mW for each; N=1024. (**a**) η≈1 bps/Hz (e.g., DCO-OFDM with 4-QAM) with $N_{\mathrm{CP}}=9$, 20, and 28, for $R_{b}=20$, 50, and 100 Mbps, respectively; (**b**) η≈2 bps/Hz (e.g., DCO-OFDM with 16-QAM) with $N_{\mathrm{CP}}=5$, 11, and 20, for $R_{b}=20$, 50, and 100 Mbps, respectively.

Since the limited LED DR constrains the transmit power, a rational solution for increasing the link span is to use multiple LEDs by putting them into an array, thus increasing the effective transmit optical power [9,53]. Consider 100 LEDs at the Tx, modulated simultaneously, and set the transmit power per LED to $P_{\mathrm{Tx},e}=50$ mW (this way, the total transmit electrical power is 5 W). We assume that we have almost the same radiation pattern (i.e., Lambertian with m≈45) as for a single LED. Figure 12 shows the BER plots for the three considered data rates and for spectral efficiencies of η≈1 bps/Hz (i.e., with 4-QAM for DCO-OFDM) and η≈2 bps/Hz (i.e., with 16-QAM for DCO-OFDM). The LBI and $Z_{\max}$ corresponding to a target BER of $10^{-3}$ are summarized in Table 6, which are larger than those for the case of a single LED (compare with Table 3 and Table 5). Nevertheless, the minimum operational distance (before SiPM saturation) is also increased, as expected [9]. For instance, for $R_{b}=20$ Mbps with η≈1 bps/Hz, the LBI is about 93.7, 91.2, and 89.3 m for DCO, ACO, and LACO schemes, respectively.

In practice, if the channel parameters (including the link range) are known at the Tx, the number of actually activated LEDs can be adjusted so as to allow working within the LBI adaptively and, hence, to optimize the link performance.

### 6.8. Impact of Bias Selection for DCO-OFDM

**Figure 13:**
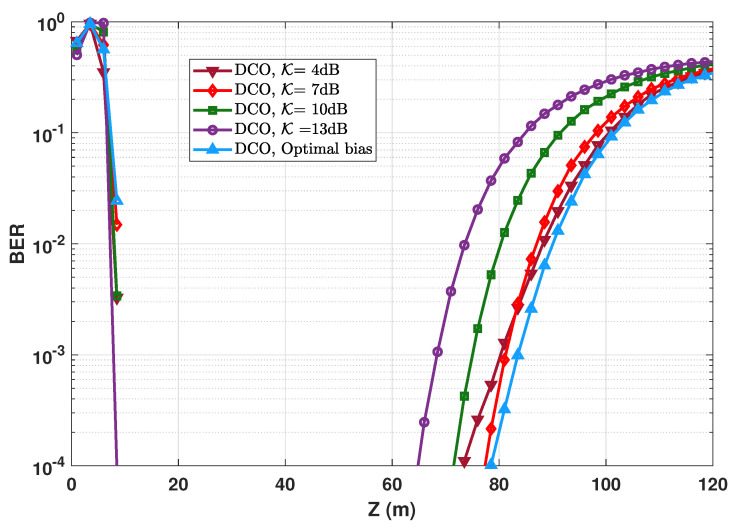
BER performance of DCO-OFDM using optimized and non-optimized DC bias. One LED at the Tx with $P_{\mathrm{Tx},e}=50$ mW; N=1024; $N_{\mathrm{CP}}=9$; $R_{b}=20$ Mbps. The corresponding clipping factors are α=0.12 for the optimum bias (corresponding to $B_{\mathrm{DC}}=2.932$), and α=0.145, 0.0922, 0.062, and 0.0432 for $K_{\mathrm{dB}}=4$, 7, 10, and 13 dB (corresponding to $B_{\mathrm{DC}}=2.9279$, 2.9344, 2.9357, and 2.9377 V), respectively.

For the sake of completeness, we provide here clarification on the impact of DC bias selection for the case of DCO-OFDM signaling. For this, consider the typical case of $P_{\mathrm{Tx},e}=50$ mW, $R_{b}=20$ Mbps, and 4-QAM signal constellation. We have compared in Figure 13 the BER performance when using the optimal bias (see Section 5.5), with the case where $B_{\mathrm{DC}}$ is calculated based on considering a clipping factor, see Equation (11). It can be clearly seen that the best energy efficiency is obtained for the case of optimized bias. For $K_{\mathrm{dB}}=7$ dB, the BER performance is relatively close to the optimum bias case. Indeed, for a too low $K_{\mathrm{dB}}$, the performance is limited by the clipping noise, whereas for a relatively large $K_{\mathrm{dB}}$, the system suffers from a poor energy efficiency due to using a too large bias. We have provided in Appendix B a more detailed analysis of the link performance when using the optimal bias and that based on $K_{\mathrm{dB}}=7$ dB.

Although the interest of optimal bias setting is obvious, its calculation (which is done numerically, see Appendix A) needs a high precision (and therefore, a high computational cost), which is explained in detail in Appendix C. Therefore, in the case of changing transmit power, for instance, in an adaptive UWOC transmission system, where the bias needs to be adjusted dynamically (based on the estimated channel state information), it would be preferable to fix the bias simply by setting the clipping factor.

## 7. Discussions and Conclusions

For the case of an SiPM-based UWOC system and in order to increase the link data rate, this work investigated the use of high spectral efficiency modulation schemes based on O-OFDM, namely the three schemes of DCO-, ACO-, and LACO-OFDM. We discussed the limitations in terms of the SiPM-based Rx saturation and the limited Tx DR, which determine the range of operation of the UWOC link for a given target BER. Indeed, the novelty of our study is that we take into account the required DC bias at the Tx for signal transmission, based on typical and practical LED characteristics. The presented results provide a reliable performance comparison of the considered schemes.

### 7.1. Main Conclusions

We showed that for moderate spectral efficiencies (namely ∼1 bps/Hz), ACO-OFDM offers the best energy efficiency, i.e., it allows us to attain the maximum link range for a given transmit electrical power. For relatively high spectral efficiencies (namely larger than 2 bps/Hz), ACO will suffer from high PAPR due to the required too large constellation size. There, LACO-OFDM becomes the best choice, as it makes a good compromise between PAPR reduction (with respect to ACO) and lower required DC bias at the Tx (with respect to DCO). Indeed, a high signal PAPR can result either in a high clipping noise level at the Tx side or a constrained link range due to SiPM saturation at the Rx side. Nevertheless, relaxing the constraint of $P_{\mathrm{Tx},e}$ at the Tx, we showed that DCO-OFDM is the most flexible scheme, offering the largest LBI, thus allowing a somehow more robust link operation when $P_{\mathrm{Tx},e}$ cannot be adaptively adjusted to the changing channel attenuation.

### 7.2. Considered Assumptions

Lastly, our study was based on a set of simplifying assumptions, including negligible turbulence effect, and perfect channel estimation and time synchronization. If these assumptions are not met in practice, the effect will be more or less the same for the three considered signaling schemes. In particular, the turbulence-induced channel fading and time synchronization errors will impact the link performance in the same way for the three schemes. Nevertheless, the impact of channel estimation errors can be more important for the case of LACO-OFDM due to the successive detection used at the Rx and the risk of error propagation between layers due to imperfect channel state information, which needs further investigation.

Our on-going work concerns practical implementation of the considered transmission schemes using a dedicated laboratory test-bed to assess their feasibility for use in a real system.

## Figures and Tables

**Table 1:** Parameters of the Tx, channel, and the Hamamatsu C13366 3050GA SiPM.

Tx (LED)	Wavelength λ	470 nm
	3 dB cut-off frequency	10 MHz
	Lambertian order *m*	45
	I-V parameters ($V_{min}$, $V_{max}$; $I_{min}$, $I_{max}$)	(2.75 V, 4.03 V; 1 mA, 110 mA)
Channel	Diffuse attenuation coefficient *K*	0.08 m^−1^
Rx (SiPM)	Photon Detection Efficiency, $Y_{\mathrm{PDE}}$	40%
	Surface Area, $A_{\mathrm{PD}}$	9 mm^2^
	Dark Current Rate, $f_{\mathrm{DCR}}$	25 kHz
	Dead Time, $\tau_{d}$	68.1 ns
	No. of SPADs, $N_{\mathrm{SPAD}}$	3600
	Cross-Talk Prob., $P_{\mathrm{CT}}$	3%
	After-Pulsing Prob., $P_{\mathrm{AP}}$	0.1%
	3 dB Cut-Off Frequency	4 MHz

**Table 2:** LBI and maximum attainable range for OOK and 4-QAM DCO-OFDM transmission schemes according to Figure 5; $P_{\mathrm{Tx},e}=125$ mW, BER $=10^{-3}$.

	$R_{b}$ (Mbps)	LBI (m)	$Z_{\max}$ (m)
DCO	1	106.6	118.8
	5	92.8	105
	10	86.4	98.8
	15	82.2	94.4
	20	78.9	91.3
OOK	1	65.6	73.7
	5	51.15	59.2
	10	41.4	49.5
	15	5.1	13.2
	20	-	-

**Table 3:** LBI and maximum attainable range for DCO, ACO, an LACO-OFDM transmission schemes according to Figure 8; $P_{\mathrm{Tx},e}=50$ mW, BER $=10^{-3}$, η≈1 bps/Hz.

	$R_{b}$ (Mbps)	LBI (m)	$Z_{\max}$ (m)
DCO	20	72.5	81.4
	50	56.5	65.4
	100	40.5	49.3
ACO	20	73.8	88.6
	50	58.6	73
	100	42.8	56.3
LACO	20	71.6	85.1
	50	56.4	69.5
	100	40.6	53

**Table 4:** LBI and maximum attainable range for DCO, ACO, an LACO-OFDM transmission schemes according to Figure 9; BER $=10^{-3}$, η≈1 bps/Hz.

	$R_{b}$ (Mbps)	LBI (m)	$Z_{\max}$ (m)
DCO	20	81	95
	50	64.9	78.9
	100	48	61.8
ACO	20	73.8	88.6
	50	58.6	73
	100	42.8	56.3
LACO	20	74.3	88.8
	50	58.9	73.2
	100	43.1	56.5

**Table 5:** LBI and maximum attainable range for DCO, ACO, and LACO-OFDM according to Figure 10; BER $=10^{-3}$, η≈2 bps/Hz.

		$P_{\mathrm{Tx},e}=20$ mW		$P_{\mathrm{Tx},e}=50$ mW	
	$R_{b}$ (Mbps)	LBI (m)	$Z_{\max}$ (m)	LBI (m)	$Z_{\max}$ (m)
DCO	20	53.2	61.6	59.1	70.1
	50	43.6	51.9	49.5	60.3
	100	33.5	40.9	38	48.9
ACO	20	25.5	40.1	3.4	23.1
	50	20.8	35.2	−	−
	100	16.1	30.2	−	−
LACO	20	51.2	63.8	18.7	34.9
	50	42	54.2	18.1	34.1
	100	31.1	34	15.6	31.2

**Table 6:** LBI and maximum attainable range for DCO, ACO, an LACO-OFDM transmission schemes according to Figure 12; BER $=10^{-3}$.

		η≈1 bps/Hz		η≈2 bps/Hz	
	$R_{b}$ (Mbps)	LBI (m)	$Z_{\max}$ (m)	LBI (m)	$Z_{\max}$ (m)
DCO	20	93.7	127.5	77.5	115.5
	50	76.5	110.1	66.5	104.3
	100	58.5	91.6	53.3	90.9
ACO	20	91.2	135.4	6.3	57.9
	50	74.5	118.4	2.88	55.5
	100	57.3	99.9	−	−
LACO	20	89.32	131.8	28.3	75.1
	50	73.2	114.5	25.9	72.4
	100	55.6	95.9	23	69.3

**Figure A1:**
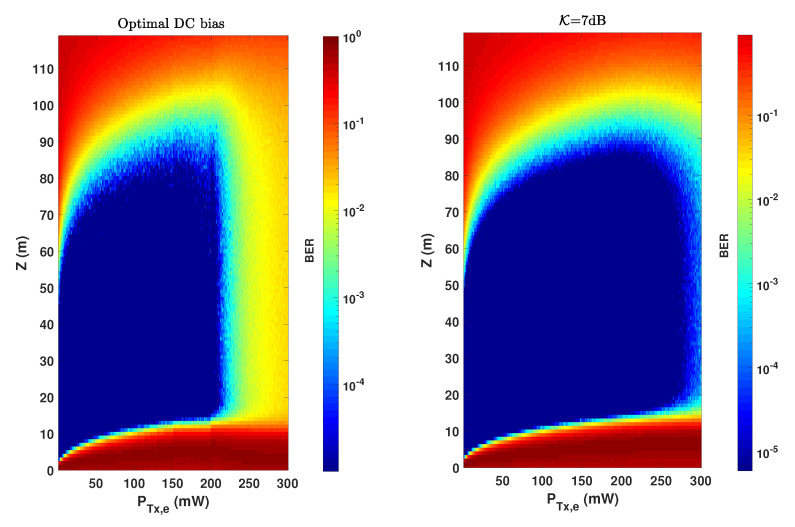
Comparison of the BER performance for DCO-OFDM: left: Optimal DC bias (HP); right: DC bias obtained by setting a clipping factor of 7 dB; DC bias obtained by setting K=7 dB but limited to 3.9 V. N=1024; $N_{\mathrm{CP}}=9$; $R_{b}=20$ Mbps, η≈1 bps/Hz.

**Figure A2:**
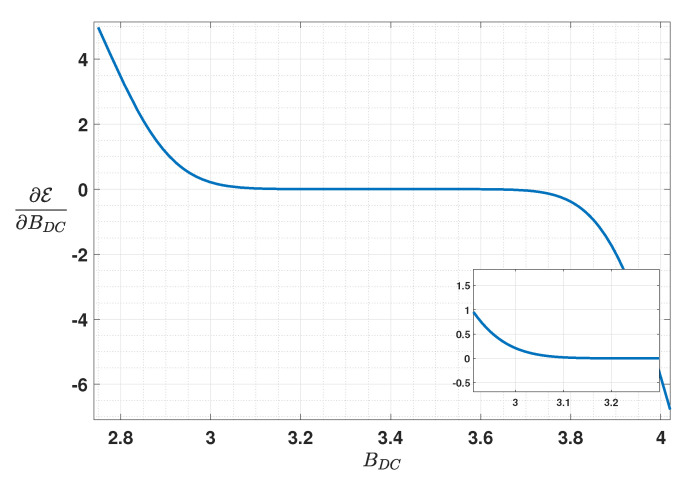
Partial derivation of E with respect to $B_{\mathrm{DC}}$ for calculating the optimal bias for DCO-OFDM. 4-QAM modulation, $P_{\mathrm{Tx},e}=50$ mW; N=1024; $N_{\mathrm{CP}}=9$; $R_{b}=20$ Mbps.

**Figure A3:**
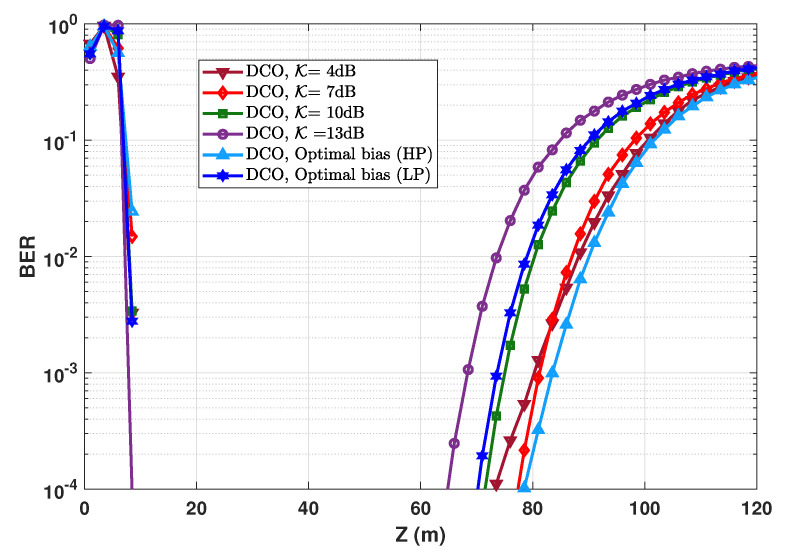
Comparison of the BER performance for DCO-OFDM obtained using low- and high-precision (LP and HP) optimal bias calculation with those calculated based on clipping factors of 4, 7, 10, and 13 dB. $P_{\mathrm{Tx},e}=50$ mW; N=1024; $N_{\mathrm{CP}}=9$; $R_{b}=20$ Mbps, η≈1 bps/Hz.

## Appendix A. Calculating the Optimal Bias for DCO-OFDM

The presented results for DCO-OFDM in the paper were mostly based on the definition of a clipping factor, for the selection of the DC bias. As explained in Section 5.5, the DC bias can be optimized in order to minimize the mean square error E between the signals before (${\overset{˘}{x}}_{n}$) and after (${\tilde{x}}_{n}$) double-side clipping, taking the LED DR into account. This appendix presents details on the optimal bias calculation.

Assuming a large enough *N*, the time-domain signal $x_{n}$ can be modeled as a zero-mean independent identically distributed Gaussian random variable with the standard deviation $\sigma_{x}$, based on the Central Limit theorem. Therefore, from Equation (12), E can be expressed by [48]

(A1) $$\begin{array}{ccc} E & = & N\;[V_{\min}^{2}\Phi \left(L\right)+\left(B_{\mathrm{DC}}^{2}+\sigma_{\overset{˘}{x}}^{2}\right)\left(\Phi (U)-\Phi (L)\right)\\ & & +2\;B_{\mathrm{DC}}\;\sigma_{\overset{˘}{x}}\left(\Phi (L)-\Phi (U)\right)+\sigma_{\overset{˘}{x}}^{2}\;L\left(\phi (L)-\phi (U)\right)\\ & & +V_{\max}^{2}\;\left(1-\Phi (U)\right)+\sigma_{\overset{˘}{x}}-2\;R_{{\tilde{x}}_{n}{\overset{˘}{x}}_{n}}], \end{array}$$

where $\sigma_{\overset{˘}{x}}$ denotes the standard deviation of $\overset{˘}{x}=\alpha x_{n}$, and $R_{{\tilde{x}}_{n}{\overset{˘}{x}}_{n}}$ is the covariance of ${\tilde{x}}_{n}$ and ${\overset{˘}{x}}_{n}$. Additionally,

(A2) $$\left\{\begin{array}{cc} U & =\left(V_{\max}-B_{\mathrm{DC}}\right)/\sigma_{\overset{˘}{x}}\;\\ L & =\left(V_{\min}-B_{\mathrm{DC}}\right)/\sigma_{\overset{˘}{x}}\;\\ \Phi (y) & =\frac{1}{\sqrt{2\pi }}\int_{-\infty }^{y}\exp\left(-u^{2}/2\right)du\;\\ \phi (v) & =\frac{1}{\sqrt{2\pi }}\exp\left(-v^{2}/2\right). \end{array}\right.$$

The optimal bias can be obtained by taking the partial derivation of Equation (A1) with respect to $B_{\mathrm{DC}}$ and setting the result to zero [48]:

(A3) $$\frac{\partial E}{\partial B_{\mathrm{DC}}}=2B_{\mathrm{DC}}\left(\Phi (U)-\Phi (L)\right)+2\;\sigma_{x}\left(\phi (L)-\phi (U)\right)=0.$$

Note that, in theory, solving Equation (A3) may result in several local minima that should be between $V_{\min}$ and $V_{\max}$. The optimum $B_{\mathrm{DC}}$ should be selected so as to result in the global minimum E from Equation (A1). Here, we calculate numerically the optimum bias from Equation (A3) for given α, $V_{\min}$, and $V_{\max}$. In addition, according to the assumption of power-normalized $x_{n}$, $\sigma_{x}=1$, and consequently, $\sigma_{\overset{˘}{x}}=\alpha$.

## Appendix B. Performance of DCO-OFDM with Optimal and Non-Optimal DC Bias

To explain in more detail the behavior for the optimal bias with increased transmit power, we have shown in Figure A1 the color maps of *Z* versus $P_{\mathrm{Tx},e}$ for the case of $R_{b}=20$ Mbps with η≈1 bps/Hz for the cases of optimal bias and classical bias setting with K=7 dB. These results require careful interpretation, as explained in the following.

For the case of optimal $B_{\mathrm{DC}}$, by increasing $P_{\mathrm{Tx},e}$, the optimal bias increases until it attains nearly the middle of the LED characteristics, i.e., $V_{\mathrm{av}}=\left(V_{\max}+V_{\min}\right)/2=3.39$ V. After this limit, if the transmit power is further increased, $B_{\mathrm{DC}}$ remains almost unchanged, but instead the scaling factor is increased to account for increased transmit power. This results in an increased clipping noise level and consequent BER deterioration. Meanwhile, fixing the maximum $B_{\mathrm{DC}}$ at $V_{\mathrm{av}}$ will make the signal be clipped “symmetrically” from upper and lower sides.

For the classical method, from Equation (11), $B_{\mathrm{DC}}$ increases steadily by increasing the transmit power. For too large $P_{\mathrm{Tx},e}$, the signal will primarily suffer from upper clipping. Meanwhile, increasing $P_{\mathrm{Tx},e}$ will result mainly in increased $B_{\mathrm{DC}}$. Hence, since α is smaller, the BER will not deteriorate as one can see for the case of optimal bias. Note that this does not mean an advantage for the classical biasing: the increased $P_{\mathrm{Tx},e}$ does not result in an improvement of $Z_{\max}$ or LBI. In other words, in the sense of energy efficiency, in either case, $P_{\mathrm{Tx},e}$ should not exceed a certain maximum transmit power.

Overall, the interest of optimal bias setting is that it results in a larger LBI and $Z_{\max}$.

## Appendix C. Required Precision for Calculating the Optimal Bias for DCO-OFDM

As explained in Section 6.8, although the optimized DC bias provides the best energy efficiency, its calculation requires high accuracy, and equivalently entails a high computational cost. This appendix clarifies this issue and shows why in practice the use of optimal DC bias could be of little interest.

Remember from Section 5.5 and Appendix A that the optimal bias $B_{\mathrm{DC}}$ is calculated so as to minimize the mean square error E. Figure A2 shows the partial derivation of $\partial E/\partial B_{\mathrm{DC}}$ as a function of $B_{\mathrm{DC}}$ considering a transmit power of $P_{\mathrm{Tx},e}=50$ mW.

Notice that the derivate is very close to zero for a large range of $B_{\mathrm{DC}}$ (between 3 and 3.8); notice also the magnified part in the figure. This signifies that the calculation of the exact optimal bias needs a high numerical precision. For instance, for the considered case study, we have calculated the optimal bias using a relatively low precision of $10^{-2}$ (the corresponding $B_{\mathrm{DC}}$ is ∼2.9406, that we will refer to as LP: Low Precision), where the resulting BER performance was worse than that with a clipping factor of 7 dB. With a higher numerical precision of $10^{-5}$ (the corresponding $B_{\mathrm{DC}}$ is ∼2.932, which we will refer to as HP: High Precision), we could attain a better performance, as can be seen from Figure A3. Although precisely calculating the bias is rather straightforward for a theoretical study, it would not be the case for a practical hardware implementation of the system with limited accuracy (e.g., using pre-registered look-up tables for a set of transmit powers $P_{\mathrm{Tx},e}$). Therefore, in this sense, the use of optimal biasing for DCO-OFDM signaling would rather have little interest in practice.
